# 

**Published:** 1908-05-16

**Authors:** 


### THE GRADUATES' MEDICAL SCHOOLS.

### Diary for the week May 18 to 23.

**LONDON SCHOOL OF CLINICAL MEDICINE, Seamen's Hospital, Greenwich, S.E.**

**At 2.15 p.m.**
**May 20,** Dr. F. Taylor, **Emphysema of the Lungs.**
**At 3.15 p.m.**
**May 22,** Mr. McGavin, **Acute Inflammation in Long Bones.**

**MEDICAL GRADUATES' COLLEGE AND POLYCLINIC, Chenies Street, W.C.**

**At 5.15 p.m.**
**May 18,** Dr. B. Abrahams, **Modern Views on Rheumatoid Arthritis.**
**May 19,** Mr. F. C. Wallis, **Fistula in Ano.**
**May 20,** Mr. A. H. Tubby, **Surgical Diseases of Children.**
**May 21,** Dr. T. G. Stewart, **Syphilitic Affections of the Spinal Cord.**

**THE HOSPITAL FOR SICK CHILDREN, Great Ormond Street, W.C.**

**At 4 p.m.**
**May 21,** Dr. Poynton, **Pyopericarditis.**

**THE POST-GRADUATE COLLEGE, West London Hospital, Hammersmith, W.**

**At 12 noon.**
**May 18,** Dr. Low, **Pathological Demonstration.**
**At 12.15 p.m.**
**May 20, 22,** Dr. Pritchard, **Practical Medicine.**
**At 5 p.m.**
**May 18,** Dr. Robinson, **Gynæcology.**
**May 19,** Dr. Low, **Insects as Carriers of Disease.**
**May 20,** Dr. Beddard, **Medicine, II.**
**May 21,** Mr. Baldwin, **Practical Surgery, I.**
**May 22,** Dr. Kenneth Scott, **Emergency Operations in Eye Affections.**

**CHARING CROSS HOSPITAL, W.C.**
Post-Graduate Demonstrations.

**At 4 p.m.**
**May 21,** Mr. Boyd, **Surgical.**

**NATIONAL HOSPITAL FOR THE PARALYSED AND EPILEPTIC, Queen Square, Bloomsbury, W.C.**

**At 3.30 p.m.**
**May 19,** Mr. Sargent, **Cerebral Abscess.**
**May 22,** Dr. J. Taylor, **Sclerotic Diseases of the Cord.**

**MOUNT VERNON HOSPITAL, Fitzroy Square.**

**At 5 p.m.**
**May 21,** Sir W. H. Allchin, **Some Inter-relations of Thoracic and Abdominal Disease.**

**THE THROAT HOSPITAL, Golden Square, W.**

**At 5.30 p.m.,**
**May 18,** Mr. Parker, **Acute Inflammatory Affections of the Nose.**
**May 21,** Mr. Parker, **Chronic Catarrhal Affections of the Nose.**

**NORTH-EAST LONDON POST-GRADUATE COLLEGE, Prince of Wales's General Hospital, Tottenham, N.**

**At 4.30 p.m.**
**May 21,** Dr. Murray Leslie, **Demonstration of Medical Cases.**

### "Printers' Pie—1908."

The illustrated annual entitled "Printers' Pie" has met with increasing popularity ever since its first edition five years ago. It is published for the purpose of increasing the funds of the Printers' Pension Almshouse and Orphan Asylum Corporation. This year's edition—published on May 13—should achieve even greater success than its predecessors. The issue consists of 150,000 copies. The contributors number 64, and among them are many of the most popular authors and artists of the day. All the illustrations are in colour, and the whole volume is excellently arranged. "Printers' Pie" is a wonderful shillingsworth.

THE HOSPITAL *May* 16, 1908.

*Name* ..........................................................

*Address* ..........................................................

..........................................................

This Coupon must accompany manuscript or contributions intended for The Hospital.

